# Surface-bioengineering of bacteriophage AP205 and MS2 virus-like particles with novel Spytag003 for antigen conjugation

**DOI:** 10.1016/j.jgeb.2026.100664

**Published:** 2026-02-04

**Authors:** Hong Liu, Ebenezer Tumban

**Affiliations:** Graduate Program in One Health Sciences, School of Veterinary Medicine, Texas Tech University, Amarillo, TX 79106, USA

**Keywords:** AP205, MS2, VLPs, Spytag003, Spycatcher003, Bio-conjugation, Immunization, Vaccine

## Abstract

The insertion of foreign antigens on the coat protein of viruses sometimes interferes with the ability of the coat proteins to assemble into virus-like particles (VLPs). To overcome this limitation, Spytag/Spycatcher bio-conjugation system was developed; however, the conjugation system has pre-existing antibodies that makes it less desirable for vaccine applications. Recently, a novel bio-conjugation system, Spytag003/Spycatcher003, was developed to by-pass this problem. The ability of this new bio-conjugation system to be used to displayed foreign antigens on VLPs has never been explored. Here we assessed whether the insertion of Spytag003 on the coat proteins of AP205 and MS2 would interfere with the ability of the coat proteins to assemble into VLPs. We showed that the insertion of Spytag003 on the N-termini of coat proteins of AP205 and MS2 did not affect their ability to assemble into VLPs. Optimized purification yielded VLPs with high purity, which were successfully conjugated with Spycatcher003 and Ag2/PRA-CSA protein. Mice immunized with the conjugated Spycatcher003 protein elicited high-titer IgG antibodies compared to immunization with unconjugated protein. However, immunization with Ag2/PRA-CSA conjugated or unconjugated to Spytag003-tagged VLPs did not have a significant difference in antibody responses between the two groups. Overall, our results show that the N-termini of AP205 and MS2 are more tolerant to Spytag003 insertions and can be used to conjugate a foreign antigen; as a proof-of-concept, we conjugated a prototype antigen, Ag2/PRA-CSA, on the VLPs. The potential to conjugate diverse antigens on these novel bio-engineered platforms should be explored further.

## Highlights

• Spytag003 insertion at the N-termini of AP205 & MS2 does not affect VLPs assembly.
• Spytag003 insertion at the C-terminus of MS2 affected expression level/solubility.
• Spytag003-tagged VLPs were conjugated with Spycatcher003 or a fungal protein.
• Immunization with Spycatcher003 displayed on VLPs elicited a balanced Th response.

## Graphical abstract

**Figure:**
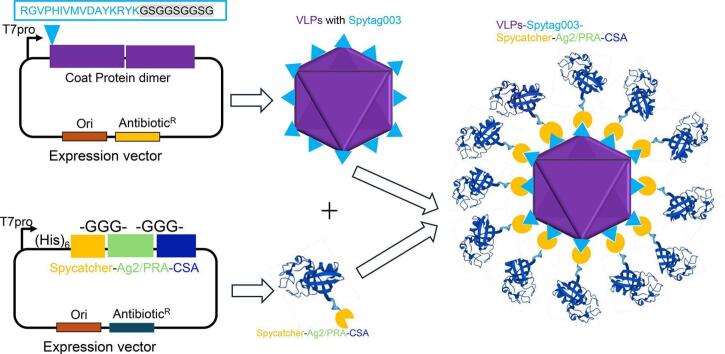

## 1 Introduction

Peptides or protein-based antigen vaccines are better alternatives to live-attenuated vaccines: i) they are very safe for everyone regardless of immune status,[1], [2], [3], [4] ii) they are very easy to produce in large quantities without a requirement for a biosafety level-3 or 4 (BSL3 or 4); higher level biosafety facilities are required to develop live-attenuated vaccines (to culture and attenuate deadly infectious pathogens),^3^ iii) the antigenic targets, especially those for peptide vaccines, are well-defined, are very specific and do not cause adverse effects; live-attenuated vaccines on the other hand have all components of the infectious agent and may be associated with reactogenicity.[5], [6] Although protein or peptide vaccines are better alternatives to live-attenuated vaccines, protein/peptide vaccines (unlike live-attenuated vaccines) are poorly immunogenic and thus, require multiple large doses (up to 400 μg) with exogenous adjuvants to enhance their responses.[2], [4], [7], [8] This is due to the fact that protein or peptide antigens are monomeric; they are not arranged geometrically into a dense-repetitive (multivalent) manner like they are on the surface of an infectious agent such as a virus.[9], [10] Additionally, peptide antigens are very unstable in serum and are rapidly degraded following immunization.[11], [12] Thus, peptide or protein antigens elicit low antibodies in addition to weak CD4 T cell responses.[9], [10] To overcome these challenges, peptide or protein antigens have been inserted or displayed on the surface of virus-like particles (VLPs) to increase their size as well as the valency or the repetitiveness of the peptide/proteins on VLPs (which are prerequisites to eliciting strong immune responses).[9], [10], [13]

VLPs are empty viral shells derived from the expression of viral structural proteins such as the capsid or envelope proteins; expressed coat proteins spontaneously self-assemble into VLPs that resemble (structurally and immunogenetically) an authentic virus except that they do not have the viral genome.[13], [14], [15] Thus, they are safe for everyone regardless of their immune status. Studies have shown that nanoparticles, such as VLPs, that are 20–200 nm in diameter can be easily taken up by antigen presenting cells (APCs) for presentation to the immune system,[9], [15], [16] on the other hand, soluble antigens such as proteins or peptides are <10 nm and thus, they cannot be easily taken up by APCs. Also, VLPs can enter the lymphatic system efficiently by direct diffusion.[9], [14] Additionally, antigens that have highly dense multivalent/repetitive structures, such as VLPs, can activate B cells (by cross-linking B-cell receptors) and induce antibody responses at much lower concentrations (5 μg) than monomeric antigens.[9], [10] Moreover, VLPs can be cross-presented in association with MHC class I; immunization with VLPs displaying T cell epitopes elicit robust protective T cell responses (both CD4 and CD8) against cancer, bacterial, and viral infections.[15], [17], [18], [19], [20] These features have made VLPs to be used as stand-alone vaccines; VLP-based vaccines have been approved against human papillomaviruses (HPVs) and hepatitis B virus.[13], [14] Unlike live-attenuated vaccines, VLP vaccines are very safe and are used in individuals with a depressed cell mediated immunity.[21], [22]

As mentioned above, VLPs have also been used as a display platform to enhance the immunogenicity of less immunogenic antigens such as peptides or proteins from other infectious agents. VLPs displaying heterologous peptides or proteins are called chimeric VLPs. The goal of chimeric VLPs is to elicit immune responses against the foreign antigen on the VLP and not necessarily against the VLP in question. We and others in the field have used VLPs as a display/delivery platform to display, multivalently, less immunogenic peptide antigens on VLPs, thus enhancing the immunogenicity of the peptides.[10], [13], [23] We have used VLPs from viruses that infect bacteria (bacteriophages or phages) to develop candidate vaccines against HPVs, Zika virus, and Chikungunya virus.[24], [25], [26] Most foreign antigens (especially peptides) that are displayed on VLPs are by genetic insertion. Unfortunately, genetic insertion is not possible with some peptide antigens including protein antigens that are >100 amino acids,^27^ the size of an antigen as well as its composition (hydrophobicity) at times can interfere with the ability of coat proteins, after expression, to assemble to form VLPs. Peptides that cannot be displayed by genetic insertion on VLPs can be displayed by chemical conjugation using a hetero-bifunctional cross-linker. The peptides in question are synthesized (by a biotech company) with a cysteine residue at either the N- or C-terminus of the peptide for conjugation to VLPs.[24], [26] While chemical conjugation seems to work most of the time, peptides with internal cysteine residues cannot be conjugated because they can form disulfide bonds with cysteine residue added at the N- or C-terminus for conjugation. This approach cannot even be used for proteins due to limitation on the size of protein that can be chemically synthesized (<165 amino acids).^28^ Overall, current approaches to display foreign antigens on VLPs have limitations that prevent the use of certain peptide or protein antigens (with important protective epitopes) for vaccine design.

To overcome the limitations above, the Spytag/Spycatcher bio-conjugation system was used to display foreign proteins on VLPs; Spytag/Spycatcher conjugation system, developed in 2012, uses a modified protein (Spycatcher, 138 amino acids protein) and short peptide (Spytag, 13 amino acids peptide) both derived from a CnaB2 domain of Streptococcus pyogenes. Mixing of the protein with the peptide allows the two to form a covalent bond with an affinity of 200 nM.[29], [30] To display foreign proteins on VLPs using this system, Spytag was inserted on VLPs and proteins for conjugation to the VLPs were expressed as recombinant proteins linked to the binding partner of Spytag (Spycatcher); the VLPs displaying the tag were mixed with the proteins linked to Spycatcher. This technology has been used to display foreign proteins on the surface of VLPs (e.g. AP205, norovirus) and to develop candidate vaccines against COVID-19, influenza, malaria, gonorrhea, etc.[31], [32], [33], [34] Nevertheless, recent studies have shown that humans have high pre-existing antibodies to the prototype Spytag/Spycatcher.^35^ Pre-existing antibodies can reduce vaccine efficacy,[36], [37], [38], [39], [40], [41] this thus make the application of the prototype bio-conjugation system less desirable. Recently, a novel bio-conjugation system, Spytag003/Spycatcher003, was developed.^42^ Spytag003 (**RGVP**HIVMVDAYK**RY**K) is three amino acids longer than the prototype Spytag (AHIVMVDAYKPTK) and differs from prototype Spytag by 6 amino acids (highlighted in bold text). Spycatcher003, the binding partner of Spytag003, differs from the prototype Spycatcher by 13 amino acids.^42^ Spytag003/Spycatcher003 reaction is 400 times faster compared to reaction with a prototype Spytag/Spycatcher. Additionally, Spytag003/Spycatcher003 requires low concentration (as low as 10 nM) of tag003/catcher003; moreover, their affinity is very high; it ranges from 21 ± 4 nM to infinity,^42^ furthermore, Spytag003 reacts with Spycatcher003 under a wide range of conditions (temp range 4–37 °C; pH range of 5–8) and buffer compositions (Tris buffered saline, tris, phosphate buffered saline, etc.).^43^ Pre-existing antibodies in the population against the novel Spytag003/Spycatcher003 is less compared to the protype conjugation system,^35^ this thus make the novel conjugation system more desirable for vaccine design. Although the prototype Spytag/Spycatcher system has already been used to conjugate foreign proteins on VLPs surface, VLPs assembly is highly sensitive to structural alterations and any modification has the potential to disrupt or alter the assembly process.[44], [45] The tolerability of the novel Spytag003/Spycatcher003 system in VLPs assembly and modification has not yet been fully explored.

In this study, we assessed the tolerability of the coat proteins of two different VLP platforms, AP205 and MS2, to the insertion of the novel Spytag003; we assessed whether the novel Spytag003 peptide (RGVPHIVMVDAYKRYK) can be inserted at the N- or C-termini of the coat proteins of AP205 and MS2 without affecting the ability of the coat proteins to assemble into chimeric VLPs. Furthermore, we assessed the potential of bio-conjugating its partner protein, Spycatcher003, to the tag on the VLPs. Additionally, we evaluated whether a prototype recombinant protein, Ag2/PRA-CSA, linked to Spycatcher003 can be bio-conjugated to the VLPs. Ag2/PRA (antigen 2 also known as proline rich antigen) and CSA (Coccidioides-specific antigen) are protective antigens that are highly expressed on the cell wall (spherule initials and spherules) of two fungi – *Coccidioides posadasii* and *Coccidioides immitis*.

## 2 Materials and methods

### 2.1 Insertion of Spytag003 to bacteriophage coat proteins and protein expression

**Fig. 1:**
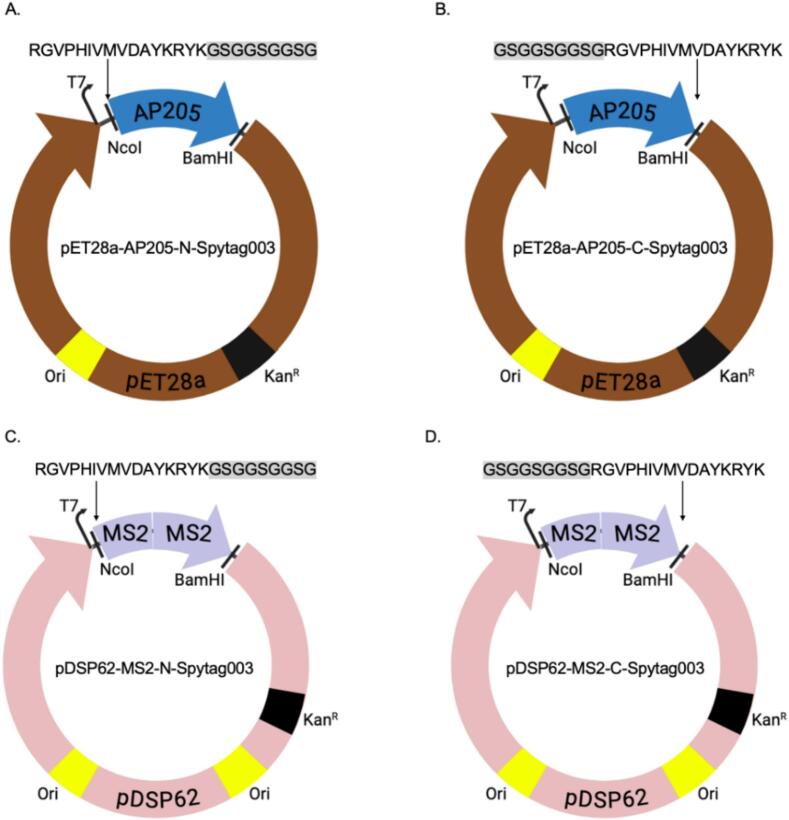
Insertion of Spytag003 at the N-terminus (A) and on the C-terminus (B) of AP205 coat protein, and insertion of Spytag003 at the N-terminus (C) and on the C-terminus (D) of the single chain dimer of MS2 coat protein. Nine amino acid linker sequence between Spytag003 and coat protein is shown in gray background.

The DNA sequence that codes for Spytag003 peptide (RGVPHIVMVDAYKRYK) was separately inserted at the N- or C-termini of the coat proteins of AP205 or that of the single-chain dimer of MS2 (Fig. 1).^46^ The coat protein of AP205 was codon optimized for *E. coli* expression and was synthesized by Epoch Life Sciences. A nine amino acid linker sequence (GSGGSGGSG) was included in-between each Spytag003 peptide insertion and the coat proteins. Four coat proteins (AP205 and MS2 each with separate Spytag003 insertions at N- or C-termini) were cloned into pET28a vectors (Epoch Life Sciences). To express the coat proteins, each construct was transformed into different strains of *E. coli* with different features: C41(DE3) which promote the expression of toxic or membrane associated proteins, Rosetta 2(DE3)pLysS which promotes the expression of heterologous proteins with rare codons in bacteria,^47^ BL21 Star(DE3) which enhances RNA stability and consequently high levels of protein expression, or ArcticExpress (DE3) which promotes the yield of soluble proteins in *E. coli***.** Individual bacterial colonies transformed with plasmids were picked from agar plates and were screened for protein expression as follows: colonies were resuspended in Luria-Bertani (LB) broth, and the cultures were grown for 3–4 h after which they were induced with 0.5 mM Isopropyl β-d-1-thiogalactopyranoside (IPTG) for 3 h. For induction of chaperone proteins in C41 cells (C41-pGro7 cells has a plasmid, pGro7, expressing chaperone proteins, groES and groEL), 0.5 mg/ml of arabinose was added to the growth media at the same time the media was inoculated with bacterial culture. The cultures were grown until an OD_600_ of 0.6. Protein expressions were induced with 0.5 mM IPTG at 16 °C and room temperature for 16 h or 37 °C for 4 h. For protein expression in ArcticExpress cells, bacteria were grown until an OD_600_ of 0.6 and protein was induced with same concentration of IPTG at 16 °C for 16 h. After the indicated time of induction, all cultures were spun down, lysed with 8 M urea and lysates were run on SDS PAGE gels. Bacterial cells that showed the highest level of protein expression were screened for solubility studies. VLPs were purified from large scale culture by lysing bacterial pellet with BugBuster® protein extraction reagent supplemented with 0.2% Triton-X 100. Bacterial lysates were centrifuged at 10,000 rpm for 10 min and supernatant were run on 23%, 29%, and 35% Optiprep gradient supplemented with 0.2% Triton-X 100. The gradient was run at 50,300 rpm (307,900×*g*) at 16 °C for 3.5 h in an SW 55 Ti rotor (Beckman Coulter). Separated layers were slowly withdrawn using a 1 mL syringe with an 18-gauge needle; the layers were run on SDS PAGE gel.

### 2.2 Expression and purification of Spycatcher003-Ag2/PRA-CSA recombinant protein

Ag2/PRA antigen (amino acids 1–106, which has been shown to offer similar levels of protection compared to the full-length sequence^48^ was fused to CSA antigen; both antigens are from *C. posadasii*. A 3-linker peptide (GGG) was included between the two proteins to increase their flexibility. The recombinant gene (Ag2/PRA-CSA) was fused to the C-terminus of Spycatcher003 gene, to enable bio-conjugation on VLPs. Six-histidine residues were added to the N-terminus of Spycatcher003 gene to enable purification of the fusion protein by affinity chromatography. The gene fragment was codon-optimized for bacterial infection and was synthesized & cloned into pET28a by Epoch Life Sciences. Protein was inducted at OD_600_ of 0.6 with 0.5 mM IPTG for 3 h. For purification, bacterial pellet was first lysed with 0.2% lysozyme solution followed by lysis with 8 M urea buffer (20 mM NaH_2_PO_4_, 20 mM Na_2_HPO_4_, supplemented with 400 mM NaCl, 50 mM imidazole, 10% tween 20, 10 mM beta-mercaptoethanol, pH 7.5). The mixture was sonicated, spun, and the supernatant added to Ni-NTA beads. The beads were washed 5X with 8 M urea buffer. Recombinant protein was eluted from the column using elution buffer (8 M urea, 20 mM NaH_2_PO4, 20 mM Na_2_HPO4, 300 mM NaCl, 250 mM imidazole, pH 7.5). Eluted protein was dialyzed and refolded by buffer exchange with refolding buffer 1 (0.5 M urea, 20 mM NaH_2_PO_4_, 5 mM reduced glutathione, 0.5 mM oxidized glutathione, 0.5 M arginine, 300 mM NaCl, 10% glycerol, pH 7.5) followed by another buffer exchange with refolding buffer 2 (20 mM NaH_2_PO4, 150 mM NaCl, 10% glycerol, pH 7.5).

### 2.3 Conjugation of Spycatcher003 and Spycatcher003-Ag2/PRA-CSA to AP205-N-Spytag003 VLPs and MS2-N-Spytag003 VLPs

Conjugation of Spycatcher003 to VLPs was done using Spycatcher003 purchased from Bio-Rad as follows: Spycatcher003 was conjugated to AP205-N-Spytag003 VLPs at 1.5:1 M ratio. To conjugate Spycatcher003-Ag2/PRA-CSA to the VLPs, purified Spycatcher003-Ag2/PRA-CSA was conjugated to AP205-N-Spytag003 VLPs and to MS2-N-Spytag003 VLPs at 1:8 M ratio and 1.7:1 M ratio, respectively. All bio-conjugation reactions were incubated at room temperature for 1.5–3 h after which they were run on an SDS PAGE gel to assess conjugation efficiency.

### 2.4 Transmission electron microscope (TEM) analysis

TEM analysis was done by absorbing VLPs on a carbon-coated glow-discharged copper grids for 2 min. The grids were negatively stained with 2% uranyl acetate for 2 min and VLPs were visualized using a Hitachi H7650 or HT7700 transmission electron microscope at different magnifications.

### 2.5 Immunization of mice

Before immunization, Optiprep was removed from conjugated VLPs using Amicon filter (50 KDa molecular weight cutoff), and buffer was exchanged to PBS. Groups of Balb/c mice (5 mice for Spycatcher003-Ag2/PRA-CSA conjugation groups and 4 mice for other groups) were immunized subcutaneously. In the first group (Spycatcher003 conjugation group), mice were immunized with a mixture of 4.2 µg of Spycatcher003 conjugated with 10.25 µg of AP205-N-Spytag003 VLPs. In groups 2 and 3 (Spycatcher003-Ag2/PRA-CSA conjugation group), mice were immunized with a mixture of 3.9 µg of Spycatcher003-Ag2/PRA-CSA conjugated with either 10.25 µg of AP205-N-Spytag003 VLPs or 1.4 µg of MS2-N-Spytag003 VLPs. Control groups received the same respective doses of the unconjugated components: Spycatcher003 (4.2 µg), Spycatcher003-Ag2/PRA-CSA (3.9 µg), AP205-N-Spytag003 VLPs (10.25 µg), and MS2-N-Spytag003 VLPs (1.4 µg). All immunizations were done twice with alum adjuvant at two-week intervals. Two weeks after the last immunizations, whole blood was collected from mice and antibody titers in sera were determined by ELISA using the above proteins as target antigens.

### 2.6 Characterization of antibodies in sera

96-well plates were separately coated with 50 ng of Spycatcher003-Ag2/PRA-CSA or Spycatcher003 overnight at 4 °C and then blocked with 0.5% milk for 2 h. Serially diluted (4-fold) sera were added (in duplicates) to the plates and incubated at room temperature for 2 h. The plates were washed with PBS, and horseradish peroxidase (HRP)-conjugated goat anti-mouse IgG, as well as HRP-conjugated goat anti-mouse IgG subclasses (IgG1, IgG2a, and IgG2b)-specific antibodies were separately added as secondary. The plates were incubated for 1 h and washed with PBS. Tetramethylbenzidine (TMB) substrate was added to the wells and incubated for 20 mins. The reaction was quenched by adding 0.1 M hydrochloric acid. Absorbance was measured at OD_450_ nm. Reciprocal of the highest serum dilution with 2-fold greater than that of control sera at the same dilution were considered as the antibody titer.

### 2.7 Statistical analysis

Statistical analyses were done using GraphPad Prism. Antibody responses were reported as geometric mean titers and Mann-Whitney *U* test was used to analyze differences in the mean values between groups. p value less than 0.05 was considered statistically significant.

## 3 Results

### 3.1 Novel Spytag003 was successfully inserted at the N-termini of the coat proteins of AP205 and MS2

**Fig. 2:**
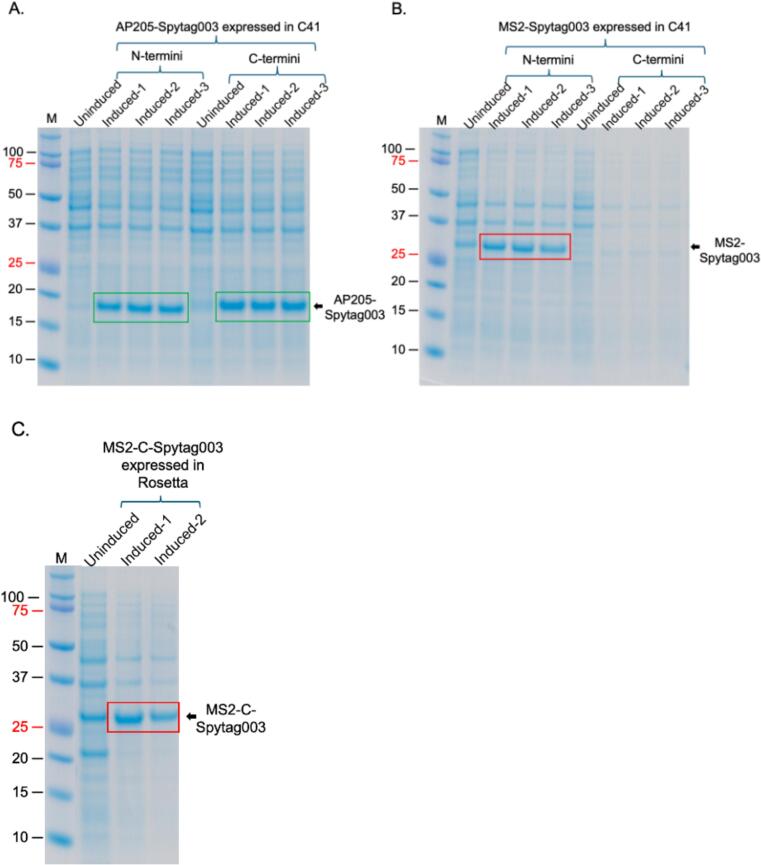
SDS-PAGE of expressed AP205 in C41 cells (A), and MS2 coat proteins with Spytag003 insertion in C41 cells (B) and Rosetta cells (C). A) Vectors expressing AP205-N-Spytag003 and AP205-C-Spytag003 were transformed into C41 cells. Cells were induced at 37 °C for 3 h with 0.5 mM IPTG, lysed with 8 M urea and were run on SDS PAGE followed by staining with Coomassie Blue. Vectors expressing MS2-N-Spytag003 and MS2-C-Spytag003 were transformed into C41 cells (B) or in BL21 Star and Rosetta cells (C). Proteins were induced and analyzed as described in panel A. M = protein marker. (For interpretation of the references to colour in this figure legend, the reader is referred to the web version of this article.)

Bacteriophage AP205 coat protein with Spytag003 insertions at both the N- and C-termini (AP205-N-Spytag003 and AP205-C-Spytag003, respectively) were successfully expressed at high levels in C41 cells (Fig. 2A). For bacteriophage MS2, only the coat protein with insertion at the N-terminus (MS2-N-Spytag003) was expressed in these cells (Fig. 2B). Given the fact that MS2-C-Spytag003 (has Spytag003 insertion at the C-terminus) was not expressed in C41 cells, we tried expressing the protein in BL21 Star and Rosetta cells. The protein was expressed in Rosetta cells (only 4 colonies after transformation) (Fig. 2C). However, no colonies were found on agar plates of BL21 Star cells transformed with plasmid expressing MS2-C-Spytag003. Out of curiosity, we transformed the cells with the vector, pDSP-MS2-N-Spytag003, that was expressible in C41 cells. There was no expression of MS2-N-Spytag003.

**Fig. 3:**
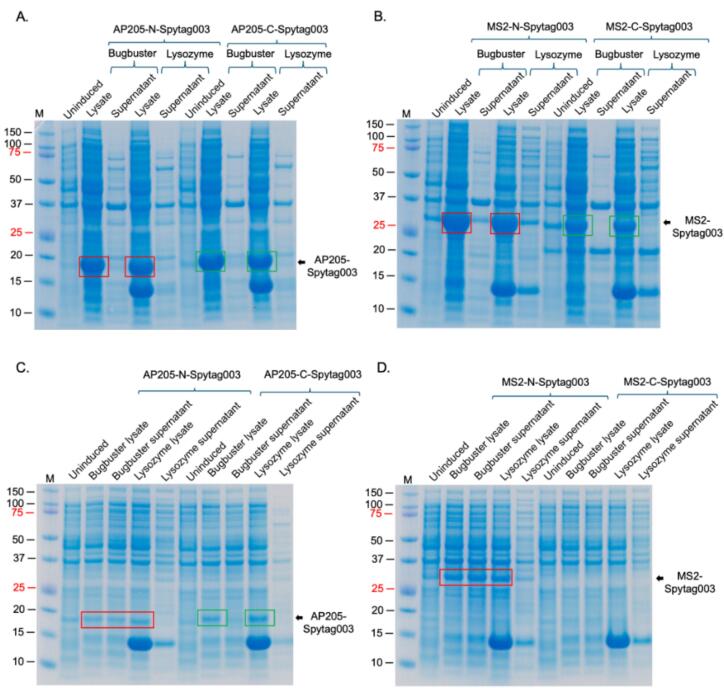

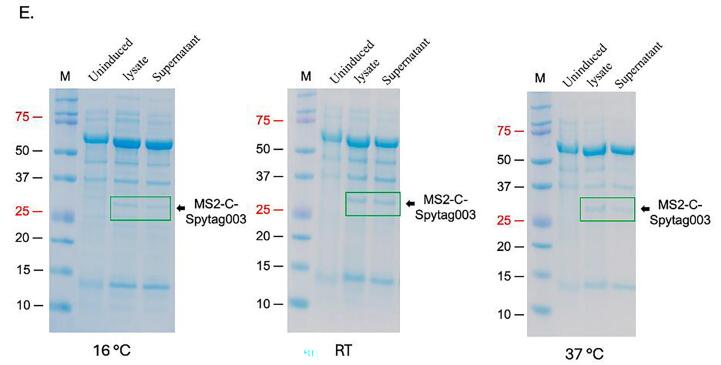
Solubility of AP205-Spytag003 and MS2-Spytag003 coat proteins. Bacterial cells expressing: A) AP205-N-Spytag003 and AP205-C-Spytag003 or B) MS2-N-Spytag003 and MS2-C-Spytag003 were induced at 37 °C for 3 h with 0.5 mM IPTG. C) AP205-N-Spytag003 and AP205-C-Spytag003 or D) MS2-N-Spytag003 and MS2-C-Spytag003 were induced at 20 °C for 16 h with with 0.5 mM IPTG. E) C41 bacterial cells expressing two chaperone proteins (groES and groEL) were transformed with expression vector expressing MS2-C-Spytag003 and expression done at 16 °C and room temperature (RT) for 16 h or at 37 °C for 4 h. groES and groEL chaperone proteins were induced as described in text with 0.5 mg/ml of arabinose. In all cases, cells were pelleted and lysed with both BugBuster protein extraction reagent and with 2% lysozyme solution; in panel E, only lysis with BugBuster was used. Lysates were spun down (10,000 rpm) and supernatants were run on SDS PAGE followed by staining with Coomassie Blue. M = protein marker. (For interpretation of the references to colour in this figure legend, the reader is referred to the web version of this article.)

To assess if the expressed proteins were soluble, for purification downstream, bacterial cells expressing the proteins above (AP205-N-Spytag003, AP205-C-Spytag003, MS2-N-Spytag003, MS2-C-Spytag003) were induced at 37 °C for 4 h; the cells were lysed with BugBuster® protein extraction reagent, or 2% lysozyme solution and supernatants were run on SDS PAGE gel. As shown in Fig. 3A and B, the proteins were not soluble when expressed at 37 °C regardless of the lysis buffer used. When the proteins were expressed at 20 °C, only the coat proteins with N-terminal insertions (AP205-N-Spytag003 and MS2-N-Spytag003) were soluble in BugBuster® protein extraction reagent (Fig. 3C and D). To assess if the proteins with C-terminal insertions (AP205-C-Spytag003 and MS2-C-Spytag003) would be soluble if expressed in different bacterial host, we transformed their expression vectors into C41 cells (C41-pGro7 cells have a plasmid, pGro7, that expresses two chaperone proteins, groES and groEL) and ArcticExpress cells. AP205-C-Spytag003 was not soluble in both cells when expressed at 16 °C (Supplemental Fig. 1). MS2-C-Spytag003 was expressed at very low levels and was soluble in C41 cells (with groES and groEL) induced at 16 °C and room temperature for 16 h or 37 °C for 4 h (Fig. 3E).

### 3.2 AP205-N-Spytag003 and MS2-N-Spytag003 formed VLPs and Spytag003 was exposed on the surface of the VLPs

**Fig. 4:**
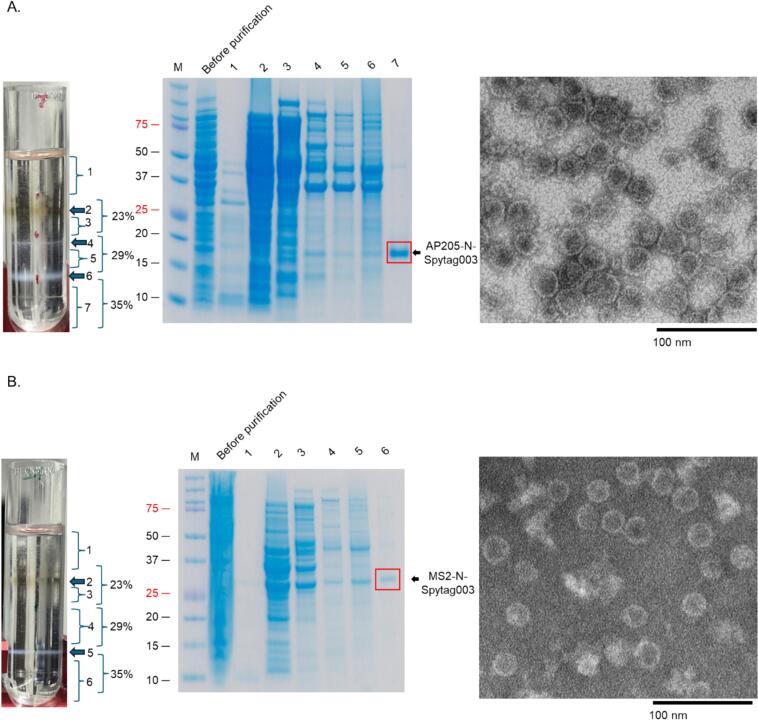
Purification, SDS-PAGE, and TEM images of AP205-N-Spytag003 VLPs (A) and MS2-N-Spytag003 VLPs (B). Bacterial supernatant expressing: A) AP205-N-Spytag003 VLPs and B) MS2-N-Spytag003 VLPs were spun on 23%, 29%, and 35% Optiprep gradient and layers (numbered 1–7; left image) were analyzed by SDS PAGE (middle) and TEM (right). TEM images were taken at 50,000× or 70,000×, respectively.

**Fig. 5:**
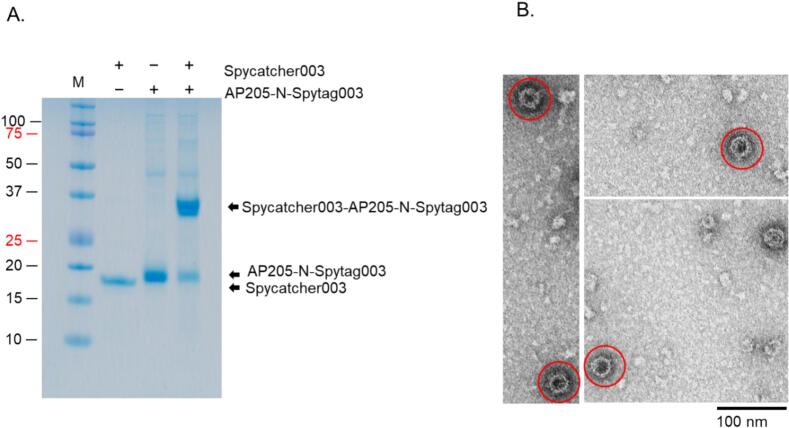
Conjugation of Spycatcher003 protein on AP205-N-Spytag003 VLPs analyzed by SDS-PAGE (A) and TEM (B). A) Spycatcher003 was conjugated to AP205-N-Spytag003 VLPs at 1.5:1 M ratio and mixture was incubated at room temperature for up to 3 h. Following conjugation, samples were run on SDS PAGE gel. B) TEM images of samples in panel A. M = protein marker. Band intensities were quantified by densitometry using ImageJ. Conjugation efficiency was calculated as follows: total coat protein before conjugation minus unconjugated coat protein, divided by the total coat protein before conjugation.

**Fig. 6:**
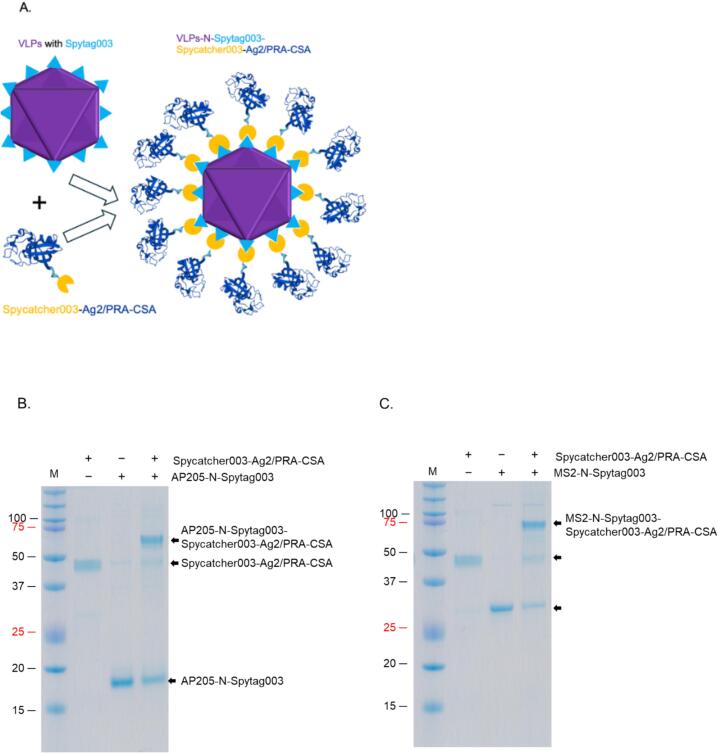

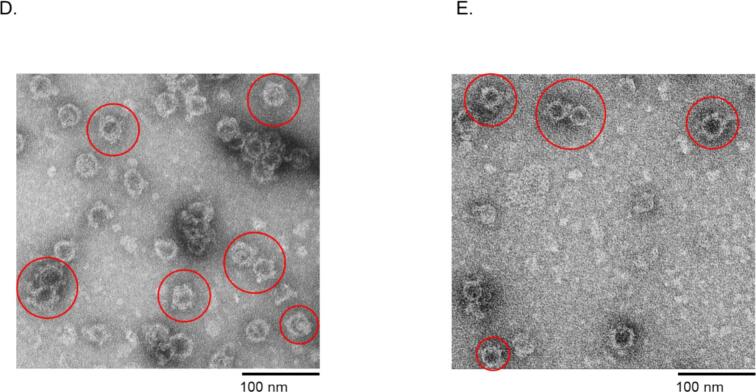
Schematic of conjugation of Spycatcher003-Ag2/PRA-CSA protein on VLPs (A) and analysis by SDS-PAGE (B-C) and TEM (D-E). Schematic in the left of panel (A) shows conjugation approach. Spycatcher003-Ag2/PRA-CSA was conjugated to AP205-N-Spytag003 VLPs (B) and to MS2-N-Spytag003 VLPs (C) at 1:8 M ratio and 1.7:1 M ratio, respectively. Following conjugation, samples were run on SDS PAGE gel. D) TEM images of samples in panel B. E) TEM images of samples in panel C. Band intensities were quantified by densitometry using ImageJ. Conjugation efficiency was calculated as follows: total coat protein before conjugation minus unconjugated coat protein, divided by the total coat protein before conjugation.

Many attempts to purify the viral coat proteins from bacterial proteins by ultracentrifugation using Optiprep were unsuccessful; the coat proteins were co-purifying with other bacterial proteins. The addition of 0.2% Triton-X 100 to lysate and buffer led to separation of the proteins, after centrifugation, into layer 7 for AP205 N-Spytag003 (Fig. 4A, left and middle images) and layer 6 for MS2-N-Spytag003 (Fig. 4B, left and middle images). Analysis of the purified coat proteins showed that the coat proteins assembled into VLPs; AP205-N-Spytag003 VLPs (Fig. 4A, right image) and MS2-N-Spytag003 VLPs (Fig. 4B, right image). To assess whether Spytag003 was exposed on the surface of the VLPs (for bio-conjugation applications), we tried conjugating Spytag003′s partner protein, Spycatcher003, to AP205-N-Spytag003 VLPs. As shown in Fig. 5, Spycatcher003 (∼15.2 KDa) was successfully bio-conjugated on the VLPs. Spycatcher003 conjugated to AP205-Spytag003 (i.e. Spycatcher003-AP205-Spytag003 complex) migrated on SDS PAGE gel at ∼34 KDa, which is close to the sum of ∼15.2 KDa (Spycatcher003) and 16.5 KDa (AP205-Spytag003); Fig. 5A. This was further confirmed by TEM (Fig. 5B). To determine if a protein can be conjugated to the chimeric VLP using Spycatcher003, we expressed and purified a prototype recombinant fungal antigen, Ag2/PRA-CSA, linked to the C-terminus of Spycatcher003 (Supplemental Fig. 2). As shown in Fig. 6, Spycatcher003-Ag2/PRA-CSA was successfully conjugated on both AP205-N-Spytag003 and MS2-N-Spytag003 VLPs indicating that Spytag003 was exposed on the surface of both VLPs. AP205-N-Spytag003 VLPs formed a complex with Spycatcher003-Ag2/PRA-CSA that migrated at ∼60.0 KDa, which is the expected size (∼16.5 KDa for recombinant VLPs + ∼43.5 KDa for Spycatcher003-Ag2/PRA-CSA) (Fig. 6B). Similarly, mixing of Spycatcher003-Ag2/PRA-CSA with MS2-N-Spytag003 VLPs gave rise to a complex that migrated at ∼73.8 KDa, which is the expected size (∼30.2 KDa for recombinant VLPs + ∼43.5 KDa for Spycatcher-Ag2/PRA-CSA) (Fig. 6C). The conjugations only took 1.5–3 h, and efficiency for AP205-N-Spytag003-Spycatcher003 was 66%, for AP205-N-Spytag003-Spycatcher003-Ag2/PRA-CSA VLPs was 40%, and for MS2-N-Spytag003-Spycatcher003-Ag2/PRA-CSA VLPs it was 68%.

### 3.3 Spycatcher003 or Spycatcher003-Ag2/PRA-CSA displayed on AP205-N-Spytag003 and MS2-N-Spytag003 VLPs elicited antibodies

**Fig. 7:**
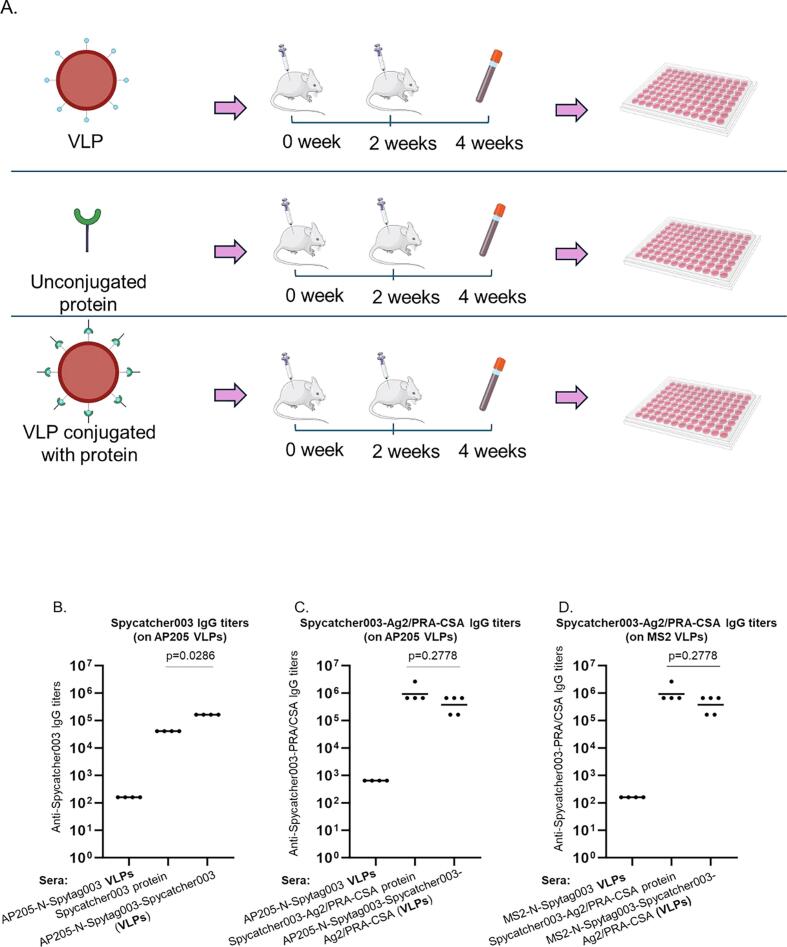
Immunization scheme (A) and ELISA of total IgG response to AP205-N-Spytag003 conjugates (B-C) and MS2-N-Spytag003 conjugates (D). Mice were subcutaneously immunized twice with the following protein or VLPs: B) 10.25 μg AP205-N-Spytag003 VLPs, 4.2 μg Spycatcher003 protein, and 10.25 μg AP205-N-Spytag003 VLPs & 4.2 μg Spycatcher003 conjugation mixture. C) 10.25 μg AP205-N-Spytag003 VLPs, 3.9 μg Spycatcher003-Ag2/PRA-CSA protein, and 10.25 μg AP205-N-Spytag003 VLPs & 3.9 μg Spycatcher003-PRA/CSA conjugation mixture. D) 1.4 μg MS2-N-Spytag003 VLPs, 3.9 μg Spycatcher003-Ag2/PRA-CSA protein, and 1.4 μg MS2-N-Spytag003 VLPs & 3.9 μg Spycatcher003-Ag2/PRA-CSA conjugation mixture. Blood was collected two weeks after the last immunization and IgG titers were determined using 50 ng of Spycatcher003 (B) or Spycatcher003-Ag2/PRA-CSA (C and D) as target antigens. Antibody responses were reported as geometric mean titers and Mann-Whitney *U* test was used to analyze differences in the mean values between groups. p value less than 0.05 was considered statistically significant.

**Fig. 8:**
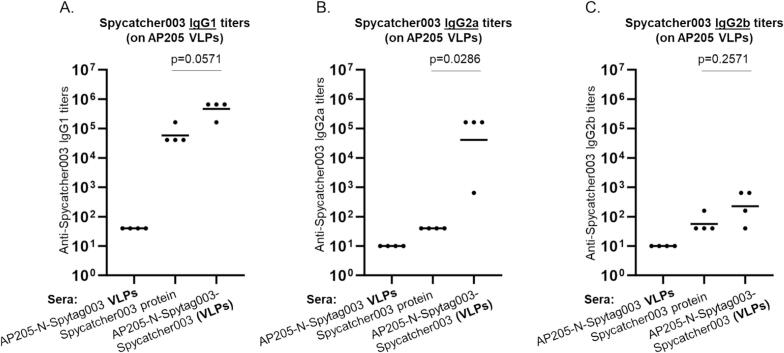
IgG1 (A), IgG2a (B), and IgG2b (C) response to AP205-N-Spytag003 conjugates. Sera from Fig. 7B above was used to do ELISA and titers were determined using 50 ng of Spycatcher003 as target antigen. HRP-conjugated goat anti-mouse IgG1 (A), IgG2a (B), and IgG2b (C) were used as secondary antibodies. Antibody responses were reported as geometric mean titers and Mann-Whitney *U* test was used to analyze differences in the mean values between groups. p value less than 0.05 was considered statistically significant.

To evaluate whether the proteins conjugated multivalently on the VLPs will elicit high-titer antibodies compared to immunization with unconjugated proteins, we immunize mice with Spycatcher003 conjugated on AP205-N-Spytag003 VLPs, with Spycatcher003-Ag2/PRA-CSA conjugated on both AP205-N-Spytag003 and MS2-N-Spytag003 VLPs (to see if there will be difference on immunogenicity of the antigen based on the two platforms), or unconjugated Spycatcher003 and Spycatcher003-Ag2/PRA-CSA for comparison (Fig. 7A). Mice immunized with only Spycatcher003 protein (without the Ag2/PRA-CSA portion) conjugated on AP205-N-Spytag003 VLPs elicited antibodies that were significantly higher than those immunized with unconjugated protein (Fig. 7B). Unexpectedly, mice immunized with Spycatcher003-Ag2/PRA-CSA conjugated on either AP205-N-Spytag003 or MS2-N-Spytag003 VLPs elicited antibodies that were slightly lower than those immunized with unconjugated Spycatcher003-Ag2/PRA-CSA protein; this difference however was not significant (Fig. 7C and D). To evaluate the IgG subtypes elicited by the conjugated and unconjugated proteins, we determined anti-Spycatcher003-Ag2/PRA-CSA and anti-Spycatcher003 IgG1, IgG2a, and IgG2b antibody titers in sera. IgG1, IgG2a, and IgG2b antibody titers were similar in trend to total IgG; in mice immunized with Spycatcher003-Ag2/PRA-CSA conjugated on either AP205-N-Spytag003 or MS2-N-Spytag003 VLPs, IgG1, IgG2a and IgG2b were slightly lower compared to those immunized with unconjugated protein (Supplemental Figs. 3 and 4). For mice immunized with Spycatcher003 protein conjugated on AP205-N-Spytag003, IgG1 and IgG2b were slightly higher compared to those in mice immunized with unconjugated protein; moreover, IgG2a was significantly higher in mice immunized with Spycatcher003 protein conjugated on AP205-N-Spytag003 (Fig. 8).

## 4 Discussion

Peptides and protein vaccines are very safe; their antigenic targets are well-defined, and they elicit a target-specific response. They are therefore better alternatives to live-attenuated vaccines, some of which are associated with reactogenicity.[5], [6] Despite the safety of peptide and protein vaccines, their immunogenicity is a problem. This can be overcome by displaying them on VLPs. Unfortunately, not all peptides or proteins can be displayed on VLPs (for reasons already mentioned above), thus making their potential to be evaluated in vaccine studies very challenging. One biggest challenge in displaying (biologically by genetic insertion) a foreign peptide or protein on the surface of a VLP is the inability of the coat protein of interest to tolerate the insertion; i.e., to assemble into a chimeric VLP.^27^ As mentioned above, prototype Spytag/Spycatcher conjugation system is compatible with AP205 and MS2 assembly,[31], [32], [33], [34] but the high pre-existing antibodies in population limits its application in vaccine design.^35^ The novel Spytag003/Spycatcher003 bio-conjugation system was developed to overcome this limitation but has never been explored on any VLPs. It is important to note that any modifications introduced into viral coat proteins can affect the ability of the coat proteins to assemble into VLPs,[44], [45] hence, it is very crucial to assess the compatibility of novel Spytag003/Spycatcher003 system with VLPs platforms.

**Fig. 9:**
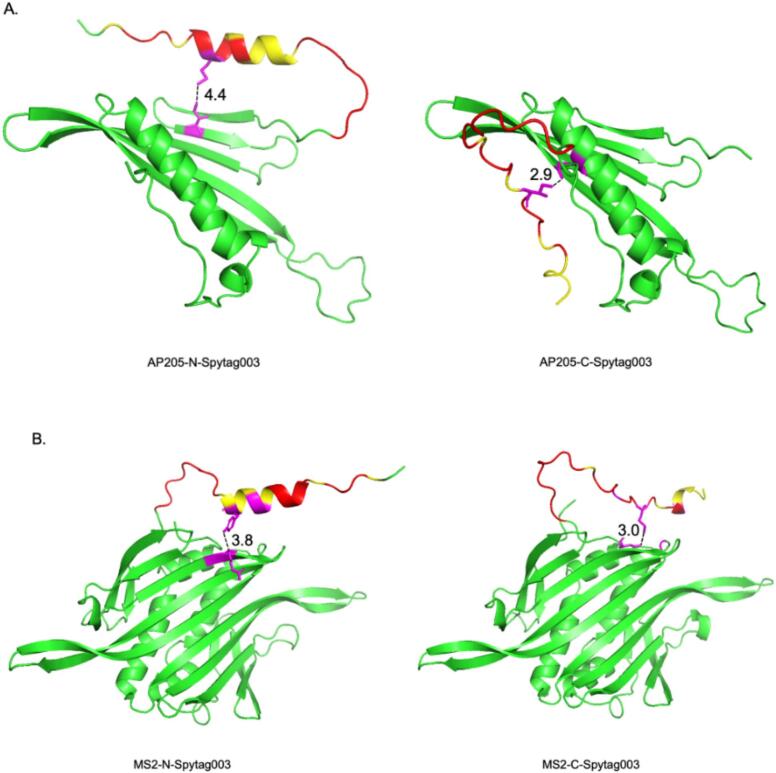
*In silico* predicted three dimensional structures of coat protein monomers with Spytag003. Coat protein sequences of AP205 (A) and MS2 (B) with Spytag003 including 9 amino acid linker were in put into AlphaFold3 program. Predicted structures were exported and analyzed using PyMOL. Closest residues between Spytag003 and coat proteins were defined using 4.5 Å cutoff; among contacting pairs, the minimum inter-residue distance was measured. Green color is AP205 or MS2 backbone. Red portion between yellow and green color is the 9 amino acid linker. Yellow color indicates hydrophilic amino acids. Purple color indicates amino acid between Spytag003 and coat proteins that are the closest to each other; residue-level closest distances are shown in numbers. (For interpretation of the references to colour in this figure legend, the reader is referred to the web version of this article.)

Here, we explored the potential to genetically insert the new Spytag003 at either the N- or C-terminus of the coat proteins of AP205 and MS2. Only the insertion of Spytag003 at the N-termini of the coat proteins of both bacteriophages were tolerated (Fig. 4). It is not clear to us why the insertion of Spytag003 on the C-terminus of the coat protein of AP205 prevented it from assembling into VLPs. A previous study had inserted the prototype Spytag peptide simultaneously at both the N- and C-termini of the VLP but not only at the C-terminus alone,^31^ thus, we speculate that the insertion at the C-terminus prevents assembly but this may be offset with an insertion at the N-terminus. As for MS2, although the same prototype Spytag has been inserted at the AB-loop of the coat protein,^27^ no attempt has ever been made to insert it at the N- or C-termini; we are also not sure why insertion of Spytag003 at the C-terminus was not tolerated especially for AP205. To gain more insight as why insertions at the C-termini of the coat proteins of both AP205 and MS2 were not tolerated compared to the N-termini, we used AlphaFold (and PyMOL for analysis) to predict (*in silico*) the structure of the proteins with Spytag003 insertions on either the N- or C-termini in comparison to the coat proteins without insertions (AP205 – PDB 5FS4; MS2 – PDB 1MSC). Side-by side comparison (structural superposition by PyMOL) of the predicted 3D structures of coat proteins with Spytag003 insertion with those without insertion showed negligible deviations between the structures. Alpha carbon root-mean-square deviation (Cα-RMSD; measures degree of similarity between 3D structures) for AP205-N-SpyTag003 VLPs was 0.21 Å; for AP205-C-SpyTag003 VLPs, RMSD was 0.25 Å, 0.28 Å for MS2-N-SpyTag003 VLPs, and for MS2-C-SpyTag003 VLPs, Cα-RMSD was 0.30 Å. These results suggest that the native core of the structures were preserved, for the most part, across all constructs and may not be the reason while the coat proteins with insertions at the C-termini were not soluble/tolerated. Thus, the observed solubility/tolerance differences were most plausibly driven by local surface interactions introduced by the SpyTag003 insertion at different termini rather than by entire misfolding of the coat proteins; the insertion of the same peptide at different sites of a coat protein has been shown to affect assembly.^49^ Based on the predictions, insertion of Spytag003 at the N-termini (regardless of whether the insertion was on AP205 or MS2) gave rise to alpha helix structure, while its insertion at the C-termini give rise to more disordered regions. Disordered regions can interfere with the assembly of macromolecular complexes^50^ and this could be one of the reasons why the insertion at the C-termini were not soluble/tolerated. Another reason can be the nature of the flexibility of the tag at the N-termini versus the C-termini. Although the N- and C-termini of the coat proteins of AP205 and MS2 are on the surface of the coat proteins (which may promote the flexibility of the insertions), analysis of the 3D structure of Spytag003 insertion at the C-termini seem to show that the space between Spytag003 and part of the coat proteins is smaller than the space between the tag at the N-termini and part of the coat proteins (Fig. 9). Measurement of residue-level closest distances between Spytag003 and coat proteins for C-termini insertions were ≤3 Å (2.9 Å for AP205-C-Spytag003 and 3.0 Å for MS2-C-Spytag003) and for N-termini insertions, residue-level closest distances ≥3.8 Å (4.4 Å for AP205-N-Spytag003, 3.8 Å for MS2-N-Spytag003) (Fig. 9). These differences in distance (especially closeness) between the tag and the coat proteins may reduce the flexibility of the tag on the coat proteins (cause crowding, especially with the C-terminal insertion) and may contribute to steric hindrance; this can affect solubility and assembly. As a matter of fact, steric hindrance has been shown to affect assembly.[51], [52], [53] Thus, experimental validation, such as circular dichroism and limited proteolysis, are needed to confirm these *in silico* predictions, which will help shed some light on why the insertions affected solubility and assembly of the recombinant coat proteins with C-terminal insertions.

Attempts to purify the chimeric VLPs by precipitation in 20%, 40% and 80% ammonium sulfate followed by size exclusion chromatography (Supplemental Fig. 5) or by ultracentrifugation using Optiprep were unsuccessful, contrasting with previous studies in which Spytag/Spycatcher modified VLPs were readily purified using these approaches.[54], [55] Coat proteins modified with functional molecules, such as Spytag/Spycatcher system, are prone to cause the aggregation of VLPs,[56], [57] but the aggregation trend of VLPs decorated with Spytag003/Spycatcher003 system has not been fully explored. Our experience revealed that Spytag003/Spycatcher003 system modified VLPs can also form aggregation. Optimization and the addition of Triton X-100 as well as careful control of the sample volume were identified as the two most critical factors in purifying the proteins from bacterial contaminating proteins. Specifically, the inclusion of Triton X-100 (0.2%) facilitated the removal of non-target proteins during purification; additionally, maintaining the sample volume no more than 30% of the total gradient volume, proved essential in minimizing co-purifying proteins with high-purity VLP-protein bands, with final purities of 96% for AP205 and 93% for MS2 (Fig. 4). This optimized protocol decreased the aggregation formation and increased VLPs recovery.

To evaluate the capability of displaying a protype protein on purified VLPs, Spycatcher003 and Spycatcher003 linked to Ag2/PRA-CSA were used for conjugation. The conjugation efficiencies for AP205-N-Spytag003-Spycatcher003, AP205-N-Spytag003-Spycatcher003-Ag2/PRA-CSA VLPs, and MS2-N-Spytag003-Spycatcher003-Ag2/PRA-CSA VLPs were 66%, 40%, 68%, respectively. This is within the range (22–88%) of AP205 conjugation with protype Spytag.^56^ On AP205 N-Spytag003 VLPs, conjugation efficiency seemed to depend on the size of the protein conjugated and the platform used; Spycatcher003 protein conjugated at a better efficiency compared to Spycatcher003-Ag2/PRA-CSA protein. Spytag003/Spycatcher003 system is stabilized by an isopeptide bond formed by lysine (Lys 31) and aspartic acid (Asp 117) side chains.^58^ The lower conjugation efficiency for Spycatcher003-Ag2/PRA-CSA on AP205-N-Spytag003 can be due to the fact that some of the conjugated Spycatcher003-Ag2/PRA-CSA on the VLPs shielded adjacent unconjugated Spytag003 tags from conjugation by more Spycatcher003-Ag2/PRA-CSA. To enhance conjugation efficiency, alternative insertion sites could be explored to display Spytag003 (if it does not affect assembly). Also, the size of the antigen used can be decreased to see if that enhances conjugation efficiency. Alternatively, pre-assembly conjugation strategy can also be explored to see if that can enhance conjugation efficiency. This requires co-expression of the Spytag003-coat proteins with Spycatcher003-Ag2/PRA-CSA.

To evaluate whether antibodies can be elicited against the conjugated proteins and the magnitude of antibodies, we immunized mice with proteins conjugated on VLPs and unconjugated proteins. Unconjugated and conjugated Spycatcher003 protein (without Ag2/PRA-CSA) were used to immunize mice to determine if Spytag003 displayed on the VLPs would elicit better antibodies compared to unconjugated protein. Immunization with Spycatcher003 protein conjugated on AP205-N-Spytag003 VLPs elicited total IgG antibody titers that were significantly higher (p = 0.0286) compared to immunization with the unconjugated protein (Fig. 7B); this data suggest that Spycatcher003 conjugated on VLPs was freely assessable to the immune system. When we looked at IgG subclasses (IgG1, IgG2a and IgG2b antibodies) we realized that mice immunized with the conjugated Spycatcher003 protein on AP205-N-Spytag003 VLPs elicited high titers of IgG1 and IgG2a (IgG2b to an extent) antibodies, compared to those immunized with unconjugated Spycatcher003 protein; the latter only elicited a high titers of IgG1 antibodies but very low IgG2a and IgG2b (Fig. 8). High levels of IgG1 are indicative of T helper (Th)2-skewed immune response while high levels of IgG2a and IgG2b are indicative of Th1-shewed response.^59^ This thus suggests that immunization with Spycatcher003 displayed on AP205-N-Spytag003 VLPs elicited a balanced Th cell immune response while immunization with the unconjugated protein elicited a Th2-skewed response. To test if the novel Spytag003 protein linked to a prototype antigen (Ag2/PRA-CSA) can elicit immune responses, unconjugated and conjugated Spycatcher003-Ag2/PRA-CSA were used for immunization. Unexpectedly, immunization with Spycatcher003-Ag2/PRA-CSA protein conjugated on either on AP205-N-Spytag003 or MS2-N-Spytag003 VLPs elicited total IgG antibodies that were slightly lower (though not significant) than immunization with the unconjugated protein (Fig. 7C and D). IgG subclasses in the group of mice immunized with Spycatcher003-Ag2/PRA-CSA did not exhibit significant difference compared to unconjugated version, too (Supplemental Figs. 3 and 4). We speculate that the concentration of Spycatcher003-Ag2/PRA-CSA protein used for immunization (3.9 µg conjugated and unconjugated format) was high enough to elicit a good immune response such that displaying it on a VLP platform had no additional benefit from the VLP, immune responses elicited at this dose are similar or superior to responses to some protein antigens immunized at high doses (>5 µg).[24], [60], [61], [62], [63] Immunization with high dose unconjugated antigen can reduce the difference in immune response caused by conjugated antigen.^54^ In addition, the enhancement of antigen immunogenicity through VLP conjugation can be protein-dependent (antigen-specific). For example, research showed that two malaria proteins, Pfs25 and VAR2CSA, were separately displayed on AP205 VLPs using Spytag/Spycatcher system; conjugated Pfs25 elicited profound immune response than unconjugated Pfs25, but IgG1 responses was higher in unconjugated group than conjugated Pfs25. With VAR2CSA antigen also displayed (separately) on AP205 VLPs, there was no difference in IgG1, IgG2a and IgG2b responses in conjugated VAR2CSA versus unconjugated VAR2CSA on VLPs,^56^ suggesting that the magnitude of responses on VLPs can be antigen-specific. This is similar to our observation with Spycatcher003 and Spycatcher003-Ag2/PRA-CSA; conjugated Spycatcher003 showed higher immunogenicity than unconjugated but that was not the case with Spycatcher003-Ag2/PRA-CSA antigen. This could be due to the fact, conjugating Spycatcher003-Ag2/PRA-CSA protein on VLPs may have caused steric hindrance or epitope masking leading to less immune responses compared to the unconjugated protein. These findings do not invalidate VLPs in general as platforms to enhance the immunogenicity of proteins, but suggest that the fusion of this particular protein, had no benefit.[64], [65] Studies are required to assess the effect of Spycatcher003-Ag2/PRA-CSA concentration and steric hindrance (conjugated vs unconjugated) on immunogenicity. Additionally, studies are required to assess the conjugation efficiency/immunogenicity of different proteins (with diverse sizes) on these platforms.

## 5 Conclusion

Overall, Spytag003 was successfully inserted to the N-termini of the coat proteins of AP205 and MS2; insertions did not affect the ability of the coat proteins to assemble into chimeric VLPs. Spytag003 insertion allowed a fungal protein, Ag2/PRA-CSA, linked to Spyachatcher003 to be conjugated to the VLPs. Conjugation reaction was conducted within 1.5 to 3 h and at lower molar ratios except for Spycatcher003-Ag2/PRA-CSA that was conjugated using 8 M. This conjugation time is faster compared to the prototype Spytag/Spycatcher system whereby some conjugations had to be done for up to 16 h.[32], [56], [66] Two novel chimeric VLPs (AP205-N-Spytag003 or MS2-N-Spytag003) developed here can be used to display foreign peptides and proteins for candidate vaccine studies without the concern of pre-existing antibodies in population. Studies are required to assess the versatility of the chimeric VLPs to the display of different antigens. Additional studies are also required to assess whether a protein that is bigger in size (such as Ag2/PRA-CSA) may not need to be displayed on these two platforms to elicit a strong humoral and balanced Th cell immune response. This study expands the toolkit for VLP engineering and provides a foundation for further applications, such as multivalent display and candidate vaccine design.

## CRediT authorship contribution statement

**Hong Liu:** Writing – review & editing, Writing – original draft, Methodology, Investigation, Formal analysis, Data curation, Conceptualization. **Ebenezer Tumban:** Writing – review & editing, Writing – original draft, Supervision, Project administration, Methodology, Investigation, Funding acquisition, Formal analysis, Conceptualization.

## Declaration of competing interest

The authors declare that they have no known competing financial interests or personal relationships that could have appeared to influence the work reported in this paper.
