# Supplementary material for: Surface-bioengineering of bacteriophage AP205 and MS2 virus-like particles with novel Spytag003 for antigen conjugation
Source: J Genet Eng Biotechnol. 2026 Feb 4;24(1):100664. doi: 10.1016/j.jgeb.2026.100664 (PMC12919298; doi:10.1016/j.jgeb.2026.100664)
Supplement: Supplementary Data 1 [file mmc1.docx]

**Supplemental information**

**
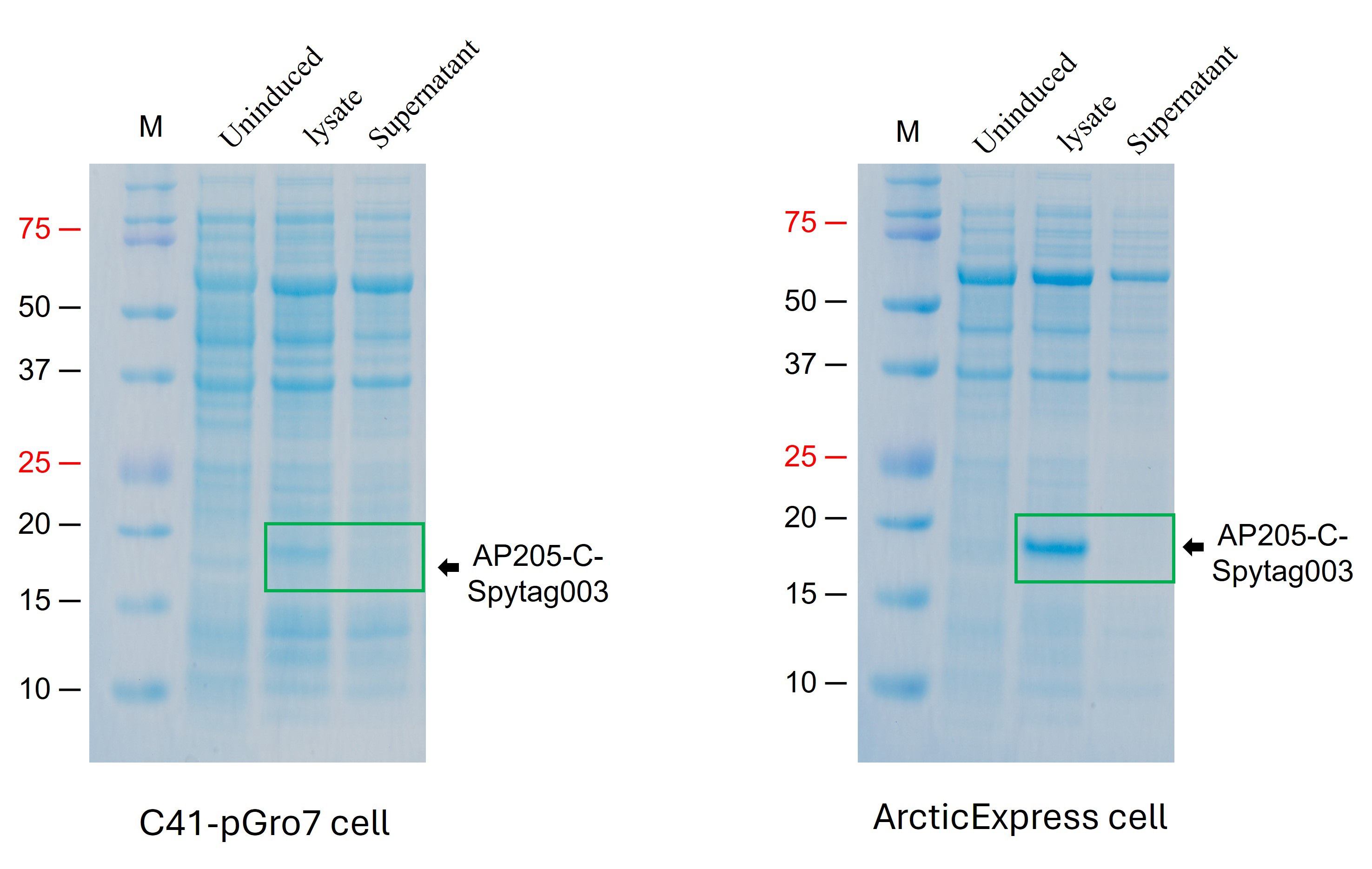
**

**Supplemental Figure 1**: Expression and solubility of AP205-C-Spytag003 in C41 cells (expressing groES and groEL) and ArcticExpress (DE3) cells. In C41 cells, expression was done at 16 °C for 16 hours with 0.5 mM IPTG. groES and groEL chaperone proteins were induced as described in text with 0.5 mg/ml of arabinose. Expression in Arctic cells was done at 16 °C for 16 hours with 0.5 mM IPTG. M = protein marker


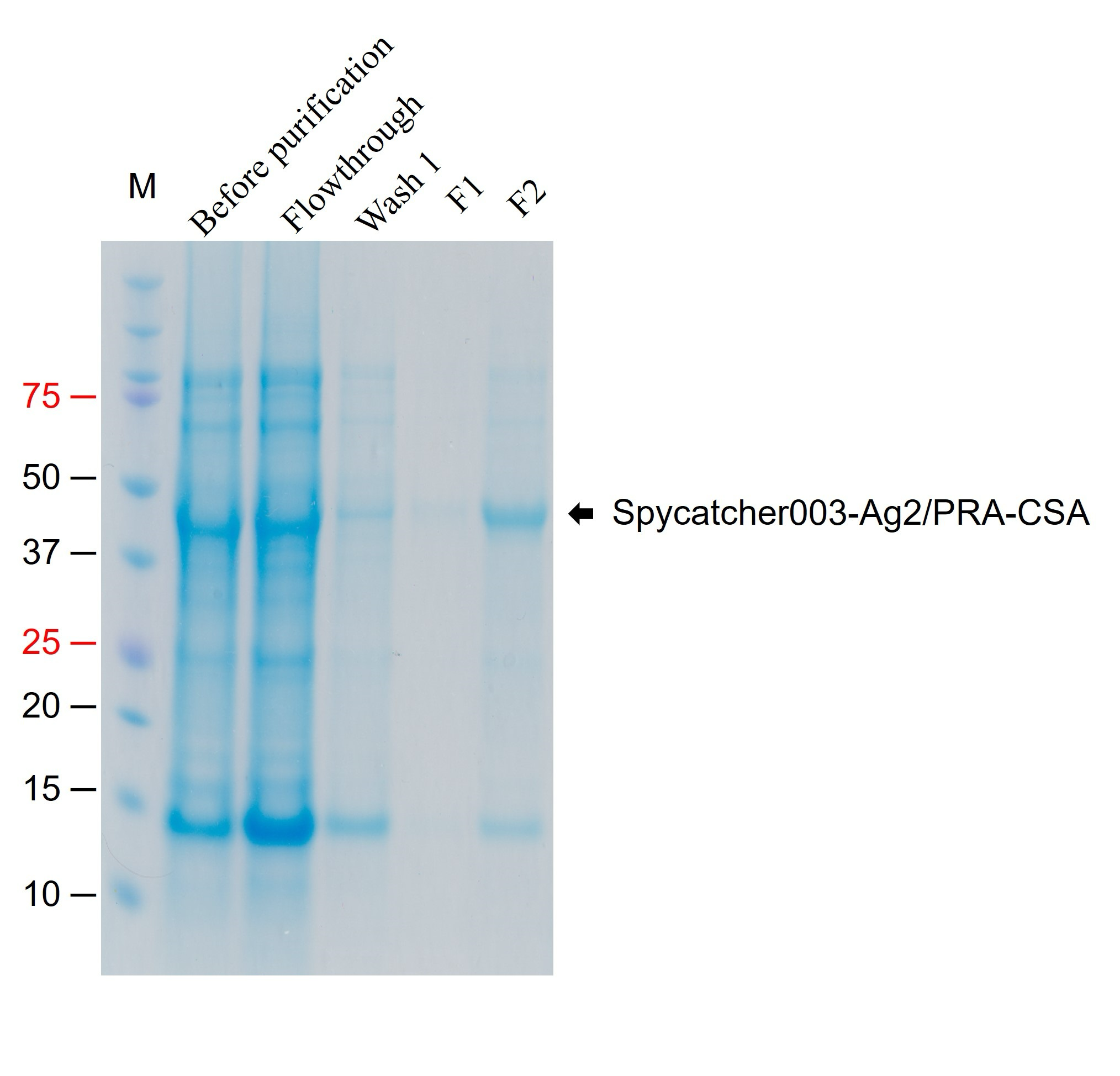


**Supplemental Figure 2**: Expression and purification of Spycatcher003-Ag2/PRA-CSA. Samples were added to Ni-NTA beads, the beads were washed and protein eluted. Fractions (F1, F2) were collected and analyzed on SDS PAGE gel. M = protein marker

**
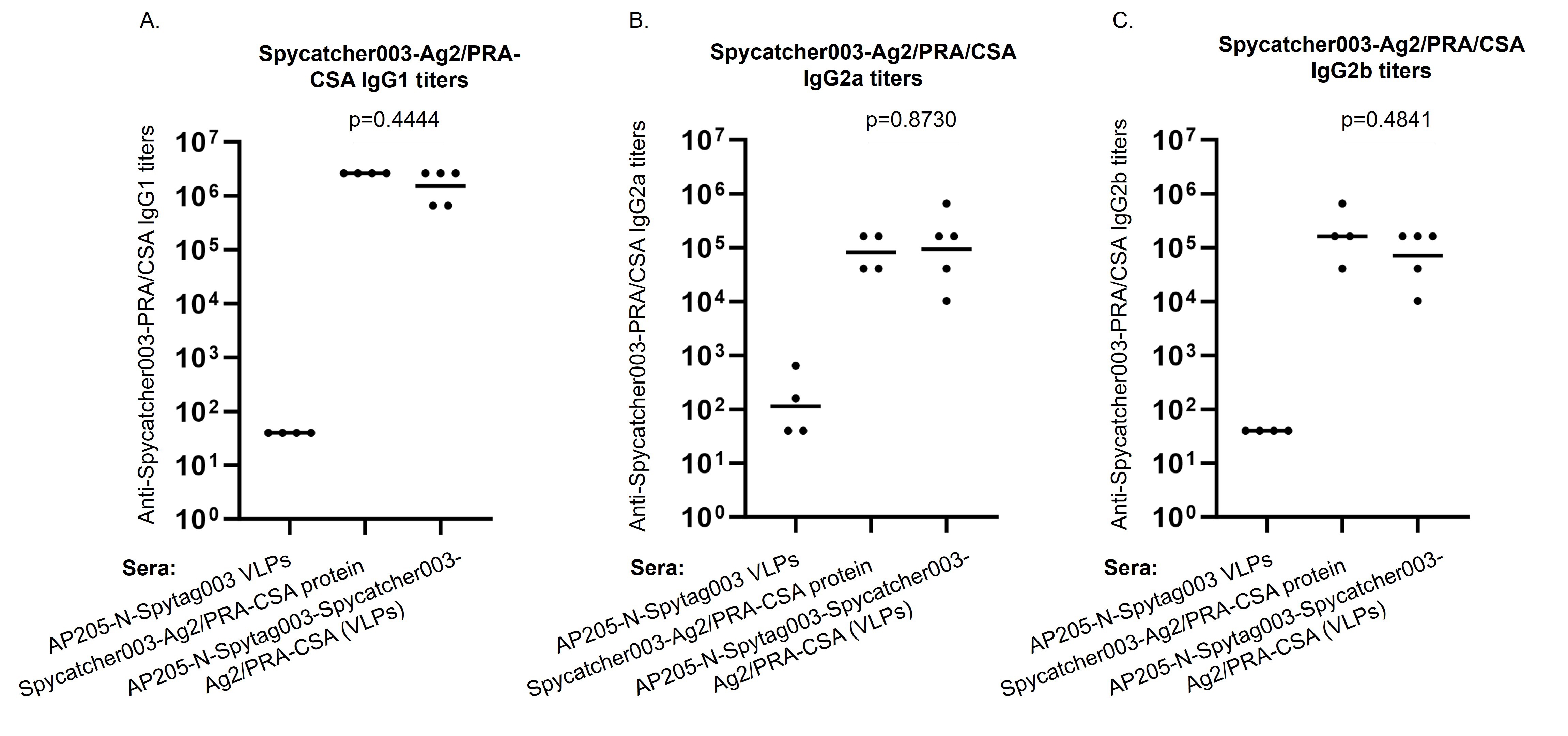
**

**Supplemental Figure 3**. IgG1, IgG2a, and IgG2b response to AP205-N-Spytag003 conjugates. Antibody responses were reported as geometric mean titers and Mann-Whitney U test was used to analyze differences in the mean values between groups. p value less than 0.05 was considered statistically significant

**
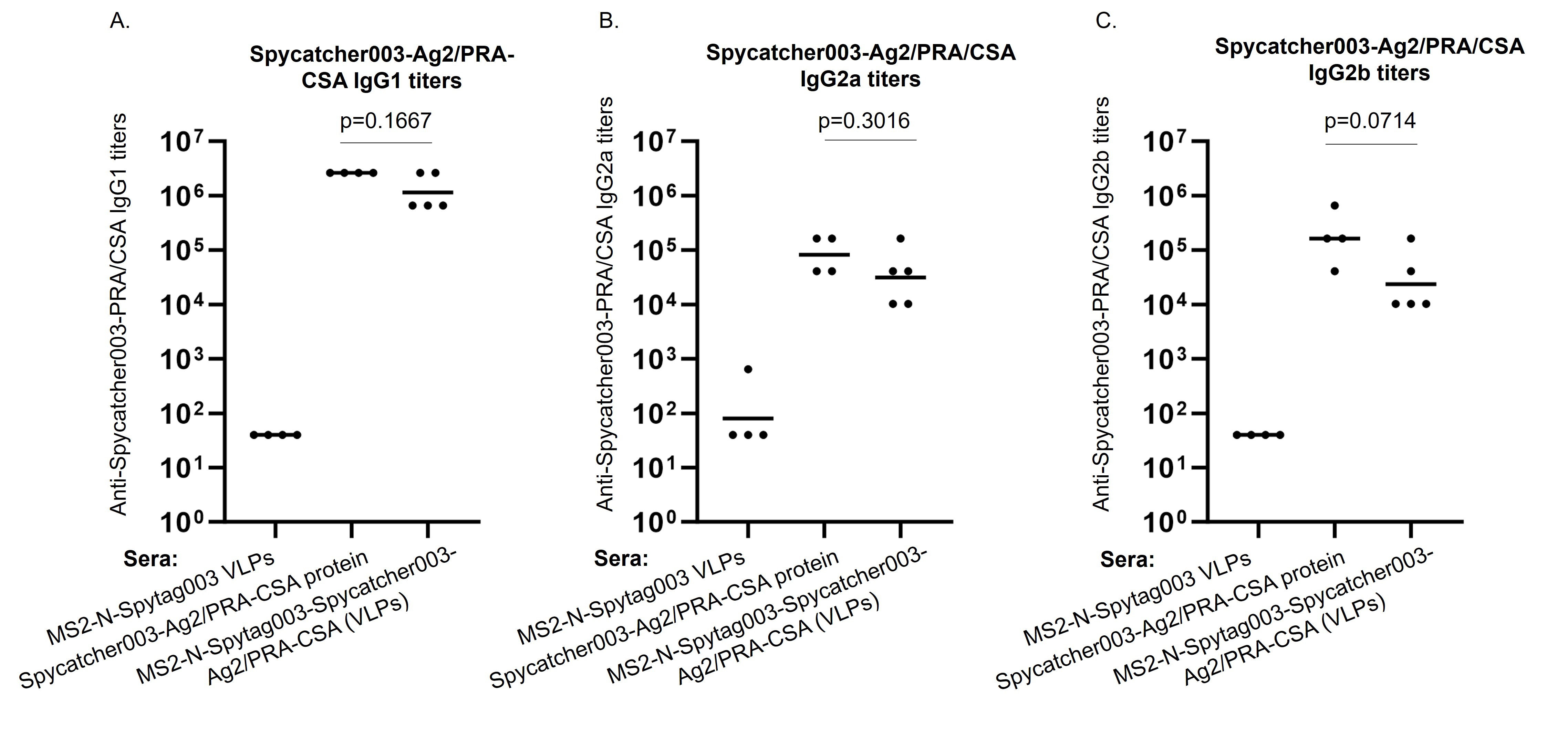
**

**Supplemental Figure 4**. IgG1, IgG2a, and IgG2b response to MS2-N-Spytag003 conjugates. Antibody responses were reported as geometric mean titers and Mann-Whitney U test was used to analyze differences in the mean values between groups. p value less than 0.05 was considered statistically significant

**
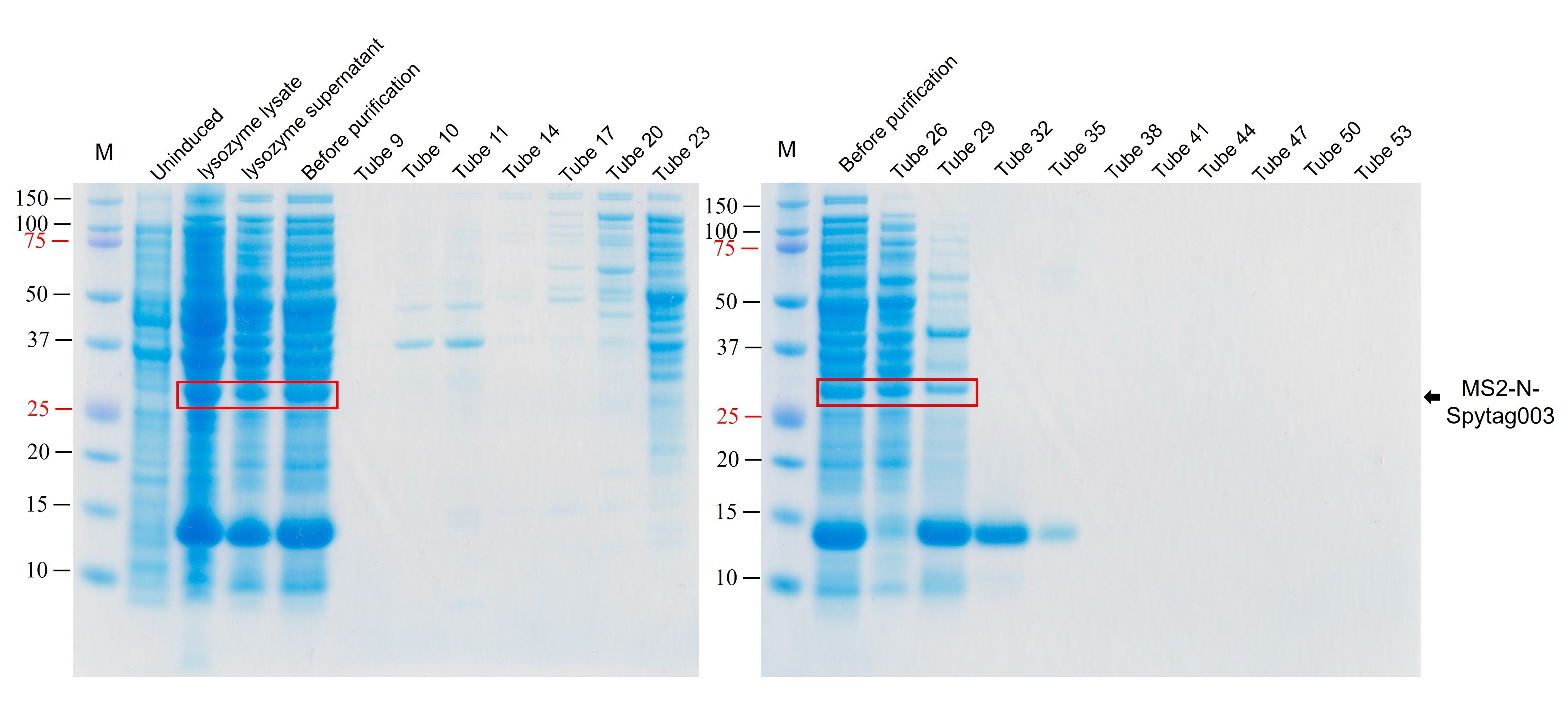
**

**Supplemental Figure 5**. Purification of MS2-N-Spytag003 VLP by size-exclusion chromatography. Samples were load on Sepharose CL-4B column and fractions were collected and analyzed on SDS PAGE gel. M = protein marker
